# Update on the Pathophysiological Role of Intracellular Signaling Pathways in Atherosclerotic Plaques and Ischemic Myocardium

**DOI:** 10.2174/157436212800376663

**Published:** 2012-05

**Authors:** Fabrizio Montecucco, Vincent Braunersreuther, Giorgio Luciano Viviani, Sébastien Lenglet, François Mach

**Affiliations:** 1 Division of Cardiology, Foundation for Medical Researches, Department of Medical Specialties, University of Geneva, Geneva, Switzerland; 2 Department of Internal Medicine, Adult Diabetes Centre, University of Genoa, Italy

**Keywords:** Kinases, plaque vulnerability, leukocytes.

## Abstract

Acute atherosclerotic complications, such as myocardial infarction, are often provoked by the rupture of an atherosclerotic plaque and the subsequent thrombotic occlusion of the arterial lumen, which interrupts the blood flow and renders ischemic the downstream peripheral tissue. Several inflammatory mediators (including cytokines, chemokines and matrix metalloproteases) have been shown to orchestrate common pathophysiological mechanisms regulating both plaque vulnerability and myocardial injury. In particular, the selective activation of certain protective intracellular signaling pathways might represent a promising target to reduce the dramatic consequences of an ischemic cardiac event. In the present review we will update evidence on the active role of intracellular kinase cascades (such as mitogen-activated protein kinases [MAPKs], Akt, Janus kinase [JAK]-signal transducer and activator of transcription [STAT]) to reduce the global patient vulnerability for acute myocardial infarction.

## INTRODUCTION

Acute cardiac ischemic diseases, such acute myocardial infarction, have been estimated to become the first cause of mortality in the worldwide population before 2030 [1]. On the basis of these catastrophic projections, the scientific community will attempt to clarify the pathophysiological processes underlying the acute rupture of atherosclerotic plaques and prevent injury in the peripheral tissue. Since atherosclerosis has been largely defined as a systemic low-grade inflammatory disease [2], different research strategies, targeting not only the “local” plaque instability, but also the systemic and “peripheral tissue” vulnerabilities, will be implemented [3, 4]. This “global” approach is expected to markedly improve the assessment of the risk and prognosis of an acute ischemic cardiovascular disease. In fact, when considered in a single issue, three determinants of cardiovascular vulnerability (involving the plaque, blood and peripheral tissue) have been proposed to possibly predict the severity of acute myocardial infarction and their sequelae [3, 4]. In humans, several soluble inflammatory mediators, such as acute phase reactants (C-reactive protein [CRP]), cytokines (tumor necrosis factor [TNF]-alpha or interleukin [IL]-6), chemokines (CCL2 and CCL5) and matrix metalloproteinases ([MMPs], MMP-2, MMP-9), have been shown to be directly associated with both plaque and systemic vulnerabilities [5-11]. Although the expression of these mediators has been widely confirmed in atherosclerotic plaques and circulating blood [4, 6], recent studies support them to play a potential “dual” role as intraplaque or systemic factors in atherogenesis [12]. On the basis of this multi-task approach, recent studies also focused on soluble inflammatory mediators to potentially increase the myocardial tissue resistance to the ischemic insult [13]. For instance, TNF-alpha (considered as a pro-inflammatory mediator increasing both plaque and blood vulnerabilities), has been shown to protect cardiomyocytes during the development of post-infarction heart failure [14]. Since TNF-alpha-mediated beneficial effect was observed at low concentrations [15, 16], these studies contributed to dramatically mitigate the ancient dichotomous concept (injury vs. protection) in atherosclerosis. We believe that it is time to become more “fluid” in exclusively considering biomarkers (reflecting the global vulnerability) as “good” or “bad” guys. Intriguingly, a single mediator (i.e. TNF-alpha) might activate opposite pathways within the plaque, the blood or the injured myocardium, thus supporting a not univocal role for this cardiovascular risk factor [14]. Therefore, in the next decade, the complex balance between pro- and anti-atherosclerotic mediators will probably shift to a matter of doses, timing, districts and signaling pathways. 

## INTRACELLULAR PATHWAYS ARE ACTIVATED WITHIN ATHEROSCLEROTIC PLAQUES AND THE ISCHEMIC MYOCARDIUM

A limited number of studies investigated the role of intracellular signaling pathways in atherosclerotic plaque vulnerability *in vivo*. Since atherosclerotic plaques are characterized by the presence of several vascular and inflammatory cells that have not been identified yet [5, 17], it is very difficult to identify the selective intracellular signaling pathways in such heterogeneous tissues. In particular, the recent discovery of different macrophage and lymphocyte subsets has further increased the complexity of the disease pathophysiology [18, 19]. Furthermore, since the isolation of cell populations from atherosclerotic plaques remains very difficult [20], a potential *ex vivo* approach to assess the intraplaque phosphorylation of intracellular kinases still requires further improvements. Evidence from animal models prone to develop atherosclerosis and knockout for certain kinases has been recently provided [21, 22]. On the other hand, several studies in rodents have demonstrated that the selective activation of some intracellular pathways is essential for saving the infarcted myocardium during reperfusion [23]. Evidence from animal studies has also shown a crucial role of mitogen-activated protein kinases (MAPKs) in the regulation of post-infarction cardiac remodeling [24]. In the following paragraphs, we will update evidence on the specific role of the most known intracellular signaling pathways regulating both plaque vulnerability and peripheral tissue resistance to ischemic insults.

## THE ROLE OF MITOGEN-ACTIVATED PROTEIN KINASES (MAPKs) 

In mammals, MAPKs are intracellular enzymes which phosphorylate downstream proteins for transducing extra-cellular signals throughout the cytosol towads the nucleus [25]. As ubiquitous enzymes, they are known to coordinate several cell functions, such as proliferation, differentiation, motility, and survival [25]. The best known MAPKs are JNK (from 1 to 3), p38 (α, β, γ, and δ) and ERK (from 1 to 5). Although their functions need to be further investigated, other proteins (such as ERK7/8, and Nemo-like kinase [NLK]), have been recently identified and included among the “classical” MAPKs. The MAPK cascade imply the phosphorylation of both upstream and downstream intra-cellular proteins [25]. The upstream enyzymes activating MAPKs are called “MAPK kinases”. The downstream substrates include members of a family of protein Ser/Thr kinases termed MAPK-activated protein kinases (MAPKAPKs) [25]. Therefore, in this complex network, the classical MAPK (JNK, p38MAPK and ERK) might differently regulate the cardiovascular vulnerability and cardiac resistance to ischemic injury.

### c-Jun NH(2)-Terminal Kinase (JNK)

The research group of Prof. Lüscher has recently showed that mice double knockout for ApoE and JNK2 develop reduced atherogenesis as compared to ApoE single knockout [26]. Intriguingly, this effect was observed only in animals lacking JNK2 and not JNK1. This protection was also confirmed by treating mice with the selective JNK inhibitor SP600125. The molecular mechanism underlying these results was indentified in the reduced differentiation of macrophages lacking JNK2 into foam cells [26]. The role of JNK2 activation in the early phases of atherogenesis was also investigated in single JNK2 knockout animals. Differently from wild type mice under high-cholesterol diet, hypercholesterolemia did not decrease the nitric oxide (NO) release from endothelial cells and the expression of antioxidants in mice JNK2-/-. These results suggest that JNK2 plays a pivotal role on endothelial dysfunction and oxidative stress in early atherogenesis. Furthermore, this article confirmed the inhibition of JNK2 as a promising therapeutic approach to reduce atherosclerosis at different stages [27]. The potential pro-atherosclerotic and pro-inflammatory activities driven by JNK phosphorylation were also confirmed *in vitro* in human inflammatory [28] and vascular cells [29], suggesting a relevance for controlling this pathway in hyperinsulinemic states associated with atherosclerotic diseases. The JNK-dependent downstream signalization was further investigated *in vivo* in mice and *in vitro* in endothelial cells. In a mouse model of carotid arteries modified with a constrictive cuff, authors showed that a disturbed flow increased the expression of NF-κB through the activation of JNK. Similarly, they observed that disturbed blood flow up regulated *in vitro* the expression of NF-κB in endothelial cells *via *JNK signaling [30]. If the JNK downstream pathways remain largely unknown, the potential upstream activation may require the deficiency of a protein mutated in the cancer-prone disease ataxia telangiectasia (ATM). In fact, its deletion in bone marrow cells might accelerate atherosclerosis in ApoE-/- mice [31]. 

The role of JNK activation in the ischemic myocardium is considered controversial. The phosphorylation of JNK in response to the cardiac preconditioning was shown by Haq and co-workers [32], but not confirmed by Nakano and colleagues [33]. This potential pathophysiological mechanism has been differently associated with cardiomyocyte protection [32, 34]. A recent study by Kaiser and co-workers, using both knockout mice for JNK1/2 and transgenic animals over expressing MKK7 (a kinase activating JNK) in the heart, failed to clarify this issue [35]. Although systemic JNK1/2 activity was found positively associated with a reduction of myocardial injury, the selective over expression of JNK in the heart was shown to protect the myocardium from ischemia reperfusion. This paradoxical results highlighted the great complexity of JNK signaling and activation in myocardial reperfusion.

### p38 MAPK

The role of the activation of p38 MAPK pathway within the plaque appears controversial. Although evidence from p38 MAPK knockout animals is still lacking, some studies investigated more selective deletions of p38 MAPK and its pharmacological inhibition in atherogenesis. In a recent article, Tabas and co-workers investigated the role of deficiency in macrophage p38alpha MAPK in ApoE-/- mice under high-cholesterol diet. The authors found increased macrophage apoptosis, plaque necrosis and a reduction in collagen intraplaque content, suggesting the presence of more vulnerable lesions as compared to control ApoE-/- mice [36]. On the other hand, the pharmacological systemic inhibition of p38 MAPK (with the selective inhibitor SB203580) has been shown to increase pro-angiogenic cells and reduced the number of inflammatory cells in plaques of ApoE-/- mice. Confirming a crucial benefit from p38 MAPK inhibition in atherogenesis, chronic treatment with SB203580 reduced size and progression of atherosclerotic lesions [37]. The promising results of p38 MAPK inhibition in mouse atherosclerotic plaques were partially confirmed by using magnetic resonance imaging (MRI) with ultra small super paramagnetic iron oxide (USPIO) contrast agents. The uptake of these iron particles within the aortas (typically done by intraplaque resident macrophages) was attenuated by the treatment with another p38 MAPK inhibitor (SB-239063) in Angiotensin II-infused apoE-/- mice [38]. Importantly, the upstream pathways governing p38 MAPK activation were recently investigated. Mice ApoE knockout and Grb2 heterozygous (a ubiquitous protein activating downstream MAPK, such as JNK and p38 MAPK) have been shown to develop a reduced lesion formation as compared to ApoE single knockout mice [39]. *In vitro* experiments using bone marrow-derived macrophages showed that Grb2 was necessary for the activation of MAPK in response to oxidized low density lipoprotein (oxLDL) stimulation [39]. On the other hand, the potential role of the apoptosis signal-regulating kinase 1 (ASK1), known to be upstream the MAPKs and thus, called as “MAPK kinase kinase”, has been recently investigated in a knockout model of atherosclerosis. ASK1 and ApoE double knockout mice under high-cholesterol diet had accelerated atherogenesis as compared with ApoE single knockout animals. This effect was associated with a reduction in the macrophage apoptotic rate within atherosclerotic plaques. The relevance for this protein in hyperlipidemia-induced atherogenesis was confirmed by comparing atherosclerotic size in animals transplanted with ASK1 (-/-) or wild type bone marrow cells. Increased lesion size was observed in mice receiving ASK1 (-/-) bone marrow as compared to wild type. ASK1-dependent pathway might be protective in hyperlipidemia-induced atherosclerosis [22]. However, these beneficial effects in atherosclerotic size might be counterbalanced by a marked increase of macrophage apoptosis, which could favor the development of a bigger necrotic core and increase plaque vulnerability. *In vitro*, p38 MAPK activation has been shown to favor the up regulation of adhesion molecules (ICAM-1 and VCAM-1) on endothelial cells [40] and leukocyte migration towards pro-atherosclerotic chemokines [41]. Therefore, these studies confirmed the risky activities of p38 MAPK in atherosclerotic inflammation. Several evidences are available on the role of p38 MAPK activation in cardioprotection from ischemia and reperfusion injury. During the last decade, three different review articles summarized the controversial results on the role of this kinase in cardioprotection [42-44]. Although several limitations (due to the variety of inhibitors for multiple isoforms of p38 MAPK) exist [44], the inhibition of p38 MAPK pathway has been shown to improve the left ventricular matrix remodeling and cell survival after ischemic events [45, 46]. The down-stream targets within cardiomyocytes potentially improved by inhibition p38 MAPK have been indicated as the stabilization of the cytoskeleton, the improvement of mitochondria functions and reduction in gap junction permeability [47-49]. Since p38 MAPK activation during reperfusion after sustained ischemia might be characterized by a biphasic phosphorylation [50], potential dichotomous activities might be hypothesized. However, since this particular activation was also observed for other kinases (such as ERK and Akt), further studies are needed to better understand the complex connections between these redundant pathways. 

### Extracellular Regulated Kinase (ERK)

Despite huge amount of *in vitro* experiments, evidence for the role of ERK activation in atherosclerotic plaque vulnerability *in vivo* is still lacking. *In vitro*, ERK has been shown to regulate several functions of inflammatory and vascular cells in atherogenesis. For instance, ERK activation is crucial for leukocyte migration in a pro-atherosclerotic microenvironment [41, 51]. Importantly, pre-incubation with statins has been shown to reduce ERK 1/2 phosphorylation and associated pro-inflammatory functions in response to C-reactive protein (CRP) in human monocytes [51]. Confirming the pro-inflammatory relevance of ERK activation, recent findings also indicate that ERK1/2 pathway is crucial for pro-thrombotic *in vitro* activities of receptor activator of nuclear factor κB ligand (RANKL) in macrophages [52]. Accordingly, we recently showed that that ERK1/2 activation is pivotal for the neutrophilic release of matrix metalloproteases (MMPs) in response to RANKL [53]. Since MMPs are considered to increase plaque vulnerability [54], the potential role of ERK pathway in their release from other cells resident within atherosclerotic plaques has been also investigated. The activation of ERK pathway has been shown to regulate the expression *in vitro* of MMP-9 in vascular smooth muscle cells (SMCs) in response to different pro-atherosclerotic stimuli [55, 56]. Finally, ERK phosphorylation was required to activate endothelial cells in several culture models [57-59]. All these *in vitro *studies indicated that ERK activation is essential for promoting atherogenesis and increasing plaque vulnerability. A strong agreement by the scientific community supports this conclusion. On the other hand, some controversies exist on the role of ERK pathway in cardioprotection. In normal conditions, the activation of Raf/MEK/ERK1/2 signaling in cardiomyocytes mediates cardiac hypertrophy (a major risk factor for the development of arrhythmias, heart failure and sudden death) [60]. On the other hand, ERK activation might also protect the ischemic hearts from cell death and reperfusion injury [60]. Evidence from *in vivo* studies partially confirms the beneficial role of cardiac ERK phosphorylation in acute myocardial infarction [61]. However, the entity of ERK phosphorylation does not correlate with the infarct size in mice [62], indicating that this process might be influenced by post-infarction infiltration of inflammatory cells within the infarcted myocardium. The early activation of ERK and Akt has been included in the more complex Reperfusion Injury Salvage Kinase (RISK) pathway [63], which is well-accepted for mediating cardiac protection. Downstream players of this pathway are still not clarified yet. Intracellular organelles (such as mitochondria), gap junctions and apoptotic modifications represent the most promising mechanism to identify the RISK pathway targets mediating benefits on ischemic cardiomyocytes [23].

## THE ROLE OF SERINE/THREONINE KINASE/PROTEIN KINASE B (AKT)

Other kinases, such as Akt, have been recently investigated in atherogenesis and plaque vulnerability with resulting opposite activities as compared to MAPKs. Akt (also called protein kinase B) is a family of serine/threonine kinases abundantly expressed in both cardiovascular and inflammatory cells [64, 65]. Akt activation is mediated by an upstream cascade involving phosphatidylinositol-3’ kinase (PI3-k) that generates phosphatidylinositol-3’,4’,5’-phosphate (PIP3), which in turn activates phosphoinositide-dependent kinase 1 (PDK1). This kinase directly phosphorylates Akt [66]. Controversial results have been shown by i*n vivo* and *in vitro* studies on the role of the Akt-dependent pathway in plaque vulnerability. Preliminary studies indicated that Akt activation might protect plaques from rupture. Interestingly, the blockade of Akt1 abrogates the beneficial proliferation and migration of vascular smooth muscle cells. This effect was confirmed *in vivo* in Akt1 and ApoE double knockout mice, which developped more vulnerable atherosclerotic plaques [21]. Therefore, this study suggests a protective role for Akt activation in advanced atherosclerosis [21]. Conversely, the activation of Akt in inflammatory cells was also associated with the reduction of their apoptosis and the functional abrogation of the mediators sustaining plaque inflammation [67, 68]. Surprisingly, Yancey and co-workers confirmed that the reduction in macrophage Akt phosphorylation was associated with an increase in intraplaque apoptosis in LDLR-/- mice transplanted with LRP-1-/- bone marrow [69]. Interestingly, this study suggested a paradoxical pro-inflammatory mechanism (due to the formation of a bigger necrotic core) increasing plaque vulnerability also when the function of inflammatory cells is neutralized. 

Since the activation of Akt pathway has been shown to promote macrophage migration and survival also after an acute myocardial infarction, Li and co-workers recently investigated the interesting cross-talk between inflammatory cells and cardiomyocytes during the post-infarction reperfusion in mice lacking Akt2 [70]. The infarcted myocardium from Akt2 knockout mice revealed an increased macrophage infiltration [70]. This increased inflammation was associated with larger infarct size and reduced cardiac function as compared to wild type mice [70], reflecting the confounding effect of Akt2 deficiency on cardiomyocyte injury.

While Akt1 activation has been shown to regulate physiologic cardiac hypertrophy in response to the GH-IGF-1 [71, 72], Akt2 phosphorylation is crucial to improve cardiomyocyte survival [72]. Thus, mainly Akt2 instead of Akt1 might be considered as an element of the RISK pathway. This direct beneficial effect in cardiomyocytes was particularly observed in response to myocardial infarction [72] and might related with the downstream inhibition of pro-apoptotic mediators, such as BAD, Bax and Caspase-9 [73-75]. It has been hypothesized that Akt2 activation in cardiomyocytes might also interfere with p38 MAPK and JNK pathways [76]. However, further studies are required to better clarify this issue. In fact, the inhibition of Akt1/2 was also found in cardiomyocytes cultured in the presence of a stress inducer of the endoplasmic reticulum, suggesting that this pathway might be involved in the regulation of cell dysfunction [77]. The association between ER stress and Akt inhibition was also confirmed *in vivo* in a mouse model of myocardial infarction [77]. However, the molecular mechanisms underlying this effect have to be identified.

## THE ROLE OF JANUS KINASE (JAK)-SIGNAL TRANSDUCER AND ACTIVATOR OF TRANSCRIPTION (STAT) PATHWAY

Since JAK/STAT activation is directly mediated by the homo- or hetero-dimerization of transmembrane receptors, this intracellular pathway represents one among the fastest transduction pathways for the salvage of cardiomyocytes from the sudden ischemic insults [78]. The direct phosphorylation of JAK induced by the intracellular domain of the transmembrane receptor is the main determinant of this immediate activation [78]. Once phosphorylated, JAK activate back the receptor to adapt docking sites for the cytosolic STAT protein. At this stage, JAK is able to phosphorylate STAT on the tyrosine residue (Tyr 705). Next, the phosphorylated STAT monomer undergoes to homo- or hetero-dimerization and becomes capable of dissociating form the receptor. Then, STAT is able to translocate into the nucleus for binding DNA and triggering gene expression [78]. JAK might also phosphorylate STAT in a specific serine residue (Ser 727), resulting in final transcriptional activities [78]. Depending on the specific JAK and STAT subtypes that have been identified in different tissues [79, 80], several inflammatory mediators have been shown to activate this pathway. In particular, interleukin (IL)-6, oncostatin M, and cardiotrophin-like cytokine 1 (CT-1) have been described as the most-known inducers of JAK-STAT pathway in the heart [81]. Importantly, these mediators were also capable of inducing opposite effects on cardiomyocyte survival, depending on the activation of different STAT family members. While the activation of STAT1 is believed to induce cardiomyocyte death, concomitant increase in STAT3 activity has been shown to increase cardioprotection *in vitro* and *in vivo* [82, 83]. These studies further complicate the role of JAK-STAT pathway in the cardioprotection from the ischemic injury. This finding was also confirmed by the lack of activity of the selective JAK inhibitor (AG-490) in modifying the ischemia/reperfusion injury *in vivo *[84]. Thus, although the JAK-STAT3 pathway has been included as a key component of the Survivor Activating Factor Enhancement (SAFE) pathway for cardiomyocytes [63], this univocal interpretation should be now considered as more controversial. This aspect was particularly important for clarifying the paradoxical role of certain pro-inflammatory mediators (such as TNF-alpha or IL-6) in both post-ischemic myocardial injury and plaque vulnerability [85]. In fact, pro-inflammatory cytokines have been recently shown to up regulate the expression of suppressors of cytokine signaling (SOCS) proteins (direct negative regulators of JAK/STAT pathways) in vascular smooth muscle cells (VSMCs) and macrophages *in vitro* and *in vivo* (ApoE-/- mice). The up regulation of SOCS, associated with the coherent suppression of STAT activation, has been shown to exacerbate atherosclerotic inflammation and progression [86]. In contrast with previously shown evidence on cardiomyocytes, this study indicates that pro-inflammatory mediators tend to inhibit the potential protective role of JAK-STAT pathway within atherosclerotic plaques. On the other hand, IL-6 (an inducer of vascular smooth muscle cells [VSMC] proliferation and release of leukocyte chemoattractants), has been shown to partially activate the JAK/STAT3 pathway [87]. Differently from the study by Ortiz-Muñoz and co-workers, this study suggests a potential active role of the JAK/STAT pathway in atherogenesis [87]. Since several cell subsets play a crucial activity within atherosclerotic plaques, we believe that too many confounders might interfere with the final activity (pro- vs. anti-atherosclerosis) of a single intracellular pathway. Thus, the role of JAK-STAT pathway in atherosclerotic plaque vulnerability remains uncertain.

## CONCLUSION

Intensive investigations *in vitro* and *in vivo* have been performed to better understand the selective activities of intracellular kinases in atherosclerosis. Given the great number of cell populations involved in plaque vulnerability and post-infarction cardiac injury, this approach implied as a considerable amount of work by several research groups. This strong effort resulted in the identification of certain promising candidates to selectively reduce plaque vulnerability and cardiac injury (Table **1**). Despite some controversies, MAPK, Akt and JAK-STAT pathways can actively influence both atherogenesis and cardiac damage and repair. However, the identification of a common pathway mediating beneficial effects in both conditions has to be performed. Therefore, since evidence for these proteins as promising therapeutic targets has been established only in cellular and animal models, a strong work is waiting for researchers to translate this exciting strategy to human beings. Several limitations (mainly due to the ubiquitous distribution of these proteins and high toxicity of the pharmacological kinase inhibitors) are still present and might delay the relevant therapeutic impact of this approach. Thus, we recommend as a priority to improve knowledge on more selective isoforms of these intracellular kinases and their functions in atherosclerosis.

## Figures and Tables

**Table 1.** Summary of the Principal Functions of Intracellular Pathway Activation in Atherosclerotic Plaque Vulnerability and Post-infarction Myocardium Injury

Pathway Activation	Plaque Vulnerability	Myocardial Infarction
JNK*	Increase (JNK2)	Controversial
p38 MAPK†	Potential increase	Controversial
ERK‡	Increase	Protection
Akt	Controversial	Protection (Akt2)
JAK-STAT§	Controversial	Protection, but controversial

* JNK: c-Jun NH(2)-terminal kinase.

† MAPK: mitogen-activated protein kinase.

‡ ERK: extracellular regulated kinase.

§ JAK: Janus kinase.
